# Supplementary material for: Chromothripsis during telomere crisis is independent of NHEJ, and consistent with a replicative origin
Source: Genome Res. 2019 May;29(5):737–49. doi: 10.1101/gr.240705.118 (PMC6499312; doi:10.1101/gr.240705.118)
Supplement: Supplemental Material [file supp_gr.240705.118_Supplemental_file_1.zip › contigs/annotated_contigs/DB77/contig.2.DB77_length_380_mean_cov_5.52631578947.docx]

**DB77_length_380_mean_cov_5.52631578947**

```
TATGAATTTGCCTATTCAGGTAAATGGAATCGTACAGCATTTGGCCTTTTGTCTGGCTTATTTTATTTAGCATAACGTTTTCAAGGTTC
 >chr6:119276748-119276889 + E=2e-70 p=4e-03
ATTCATGTTGTAACATATATCAGGATTTTATTTCTTTTTAAGGCTGAAT|GAC|ATGTGGCTCCATGTCCTTTCTACTGAGCAGTGATT
                                                  >chr6:119300200-119300442 + E=2e-134
GATTTCTGGGACACCTCAATGTCCTGAGTCCACTTAGTCCAAAAAGGACAGATTAGAGTACCAGTGGTGTTGCTTGCATAGCTGGAGTA

CAGAGAGAGCTGAGGGGAATTCACTTTACTGTTTGTGTCCTGCCTATTCATGTGGTAACTTCAAATAAAATATGCAGTACACTTAGATA

TTCATTTTGACGTTTCAAAATTTTAA
```
